# Individual alerting efficiency modulates time perception

**DOI:** 10.3389/fpsyg.2015.00386

**Published:** 2015-04-07

**Authors:** Peiduo Liu, Wenjing Yang, Xiangyong Yuan, Cuihua Bi, Antao Chen, Xiting Huang

**Affiliations:** ^1^Key Laboratory of Cognition and Personality, Ministry of Education, Faculty of PsychologySouthwest University, Chongqing, China; ^2^School of Educational Science, Research Center of Psychological Development and Application, Sichuan Normal UniversityChengdu, China

**Keywords:** attention, alerting efficiency, duration perception, temporal sensitivity, individual difference

## Abstract

Time perception plays a fundamental role in human perceptual and motor activities, and can be influenced by various factors, such as selective attention and arousal. However, little is known about the influence of individual alerting efficiency on perceived duration. In this study, we explored this question by running two experiments. The Attentional Networks Test was used to evaluate individual differences in alerting efficiency in each experiment. Temporal bisection (Experiment 1) and time generalization task (Experiment 2) were used to explore the participants’ perception of duration. The results indicated that subjects in the high alerting efficiency group overestimated interval durations and estimated durations more accurately compared with subjects in the low alerting efficiency group. The two experiments showed that the sensitivity of time was not influenced by individual alerting efficiency. Based on previous studies and current findings, we infer that individual differences in alerting efficiency may influence time perception through modulating the latency of the attention-controlled switch and the speed of the peacemaker within the framework of the internal clock model.

## Introduction

Perception of brief event durations is fundamental to a range of human perceptual and motor activities that include motor control in walking, speaking, playing music, driving a car, and participating in sports. Temporal illusions, in which the experience of time is not isomorphic to physical time, have long been reported in daily life as well as in psychophysical studies (Wittmann et al., 2010; Allman and Meck, 2012). For instance, emotionally aversive events are perceived to last longer than their physical duration (Droit-Volet and Meck, 2007; Wittmann and van Wassenhove, 2009; Gil and Droit-Volet, 2011; Grommet et al., 2011). Studies have also documented that stimuli with larger magnitudes, intensity, and complexity expanded perceived duration, whereas stimuli that are repeated, have high probability, and non-salient compressed time (Eagleman, 2008; van Wassenhove et al., 2008; Birngruber et al., 2014). Although the mechanisms of temporal illusions are still under debate, attention, and arousal have consistently been proven to be key factors that can influence time perception (Zakay, 1989; Block and Zakay, 1996; Zakay and Block, 1996, 1997; Droit-Volet and Meck, 2007; Merchant et al., 2013).

Attention can be divided into two aspects: selective and non-selective alerting components. The former represents the selection dimensions, i.e., focused and divided attention, whereas the latter represents the intensity dimensions, i.e., alerting and sustained attention (Posner and Petersen, 1990; Robertson et al., 1998; Sturm and Willmes, 2001; Weinbach and Henik, 2011). Most studies focused on the effects of the selection aspects of attention on time perception (Coull et al., 2004; Hemmes et al., 2004; Ulrich et al., 2006; Liu et al., 2013b; Henry et al., 2015). The internal clock model states that the higher the amount of attention focused on time, the more pulses accumulated. Empirically, people judge a certain duration to be longer when they allocate more attention to the target duration; whereas any attention that shifts from the target duration leads to shorter estimates (Treisman, 1963; Meck and Church, 1983; Church, 1984; Gibbon et al., 1984; Coull et al., 2004, 2011; Buhusi and Meck, 2005; Shi et al., 2013). However, little is known about the role of the non-selective alerting component of attention in the processing of time perception.

Alerting, a non-selective component of attention, refers to a state of general readiness that enhances processing of a stimulus and initiation of a response (Posner, 2008). There are two types of alerting; i.e., phasic alerting, which is specific to a task, and intrinsic alerting, which is general cognitive arousal (Raz and Buhle, 2006). The alerting mentioned in this study is specified as phasic alerting. This ability can maintain and increase response readiness for a forthcoming stimulus without any specific prior selection. Phasic alerting is considered as the basis for operations such as orienting and selective attention (Sturm and Willmes, 2001; Husain and Rorden, 2003; Finke et al., 2012). The paradigm often used to study phasic alerting is measuring how an infrequent, unpredictable warning signal preceding the presentation of a stimulus affect subjects’ response time (RT). Participants can maximize their alerting efficiency to prepare for the impending stimulus because of the alert of the warning signal. Previous studies have showed that when a stimulus is presented followed by a warning signal, the RT of participants is substantially faster compared to when no such warning is given (Nebes and Brady, 1993; Robertson et al., 1998; Fan et al., 2005).

Not only different alerting state has an impact on the perceptual processing speed, visual conscious perception, and time perception of children (Droit-Volet, 2003; Matthias et al., 2010; Kusnir et al., 2011; Weinbach and Henik, 2011; Finke et al., 2012), but also the different individual alerting efficiency influences human behavior (Leproult et al., 2003; Posner, 2008; DeGutis and Van Vleet, 2010). For instance, individual differences in alerting also impact the cognitive process (Liu et al., 2013a). Alerting efficiency can serve as one of the indicators in the selection of athletes, pilots, and bus drivers (Tafti et al., 1992; Brown et al., 2007; Petróczi and Aidman, 2008). To the best of our knowledge, no study has explored how individual differences in alerting efficiency influence an individual’s time perception.

We used two experiments to investigate how alerting mediated time perception, with each experiment including two tasks. The Attentional Networks Test (ANT) allows assessment of the efficiency of attentional networks involved in the distinct functions of alerting, orienting, and executive attention (Posner and Petersen, 1990; Fan et al., 2002, 2005). We evaluated the individual differences in alerting efficiency through the ANT in two experiments. In Experiment 1, the temporal bisection task was used to explore the individual differences in alerting efficiency. In Experiment 2, the time generalization task was used to further validate the reliability of findings in Experiment 1.

## Materials and Methods

### Experiment 1

#### Ethics Statement

This research was approved by the Human Research Ethics Committee of the Southwest University of China. All the participants signed an informed consent prior to the study.

#### Participants

Forty four right-handed undergraduate and graduate students (eight males; age range = 17–24 years, mean age = 21 years) took part in the study. They all had normal or corrected-to-normal vision.

#### Stimuli and Tasks

The ANT and temporal bisection task were presented using E-Prime software (Psychological Software Tools, Pittsburgh, PA, USA). The standard procedures for ANT were followed (https://www.sacklerinstitute.org/cornell/assays_and_tools/ant/jin.fan/). A fixation point of randomly determined duration (400–1600 ms) was presented at the beginning of each trial and a cue was then presented for 100 ms. There were four cue conditions; i.e., no-cue, center-cue, double-cue, and spatial-cue. The fixation point alone was presented for a randomly determined duration of 350–650 ms in the no-cue condition. Two asterisks were simultaneously presented at two points during the double-cue condition. An asterisk was presented at a target position for 100 ms in the spatial-cue condition. After the cue was presented, the fixation point was presented again for 400 ms. The target was then subsequently presented at a visual angle of 0.096∘ either below or above the fixation point. The target location varied in each condition except for the spatial-cue condition. The participants were instructed to maintain fixation on the centrally located fixation point throughout a task until the stimulus appeared. When the stimulus appeared, they were told to respond as quickly and accurately as possible. All stimuli were composed of five arrows. Response consisted of pressing the left mouse button using the left thumb when the central arrow pointed to the left. If the central arrow point to the right, they were instructed to use the right thumb to push the right mouse button. A stimulus was presented until a participant pressed a button. If the participant did not present a button within 1700 ms, the stimulus was extinguished. The stimulus disappeared immediately after a subject response, and a target fixation point was then displayed for a variable duration. The duration of the post-response target fixation point was 3500 ms minus the duration of the first fixation minus the reaction time. The participant completed practice trials before the formal tests were initiated. A total of 24 practice trials with full feedback were performed before the formal tests. The ANT formal testing protocol consisted of 96 trials. Each of the four cue conditions were performed with two target locations plus two flanker conditions plus two central letters with three repetitions.

**FIGURE 1:**
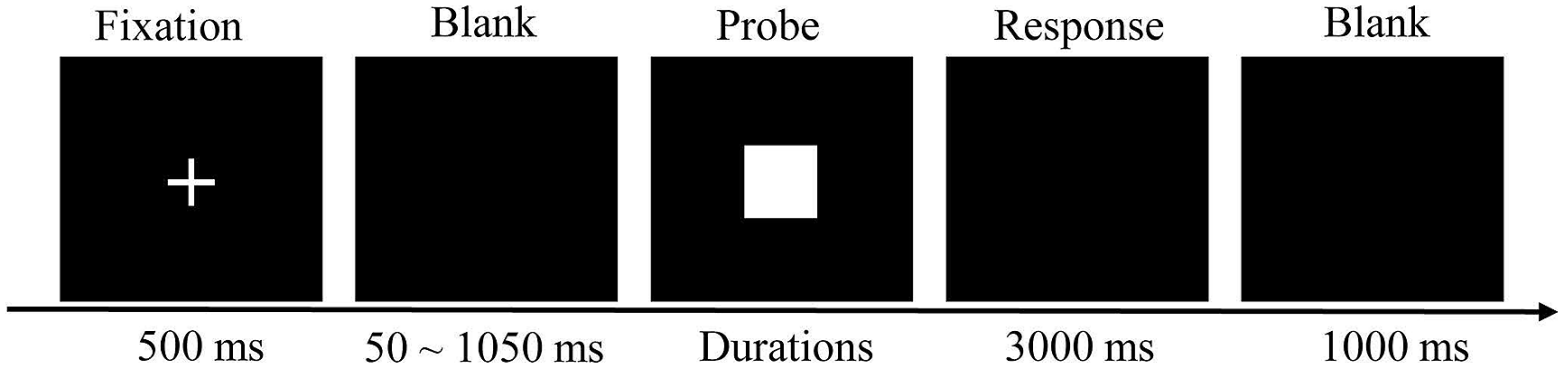
**Trial sequence of the test phase in the temporal bisection task**.

The temporal bisection task was the second part of Experiment 1. The temporal bisection task started with a training phase that consisted of “short” (700 ms) and “long” (1300 ms) anchor probe durations and a test phase that included seven probe durations (700, 800, 900, 1000, 1100, 1200, and 1300 ms). The short and long anchor duration stimuli were white squares (135 pixels wide and 133 pixels high) on a blank screen with the short and long durations counterbalanced. The task for the participant was to press a button that indicated they perceived either the short or long duration. Each session comprised 10 trials with a random presentation of five short and five long durations. The response keys were changed for different participants and were ultimately counter-balanced. A fixation point was presented in the center of the screen at the beginning of each trial for all temporal tasks. The fixation point was presented for 500 ms and then the target square was presented. This was followed by a blank screen for a randomly determined duration of 50–1050 ms (**Figure 1**). The participants pressed the response key when the screen was blank. The blank screen was presented for 3000 ms if the participants did not respond. The response indicated whether the duration of the stimulus was more similar to the short standard duration or the long standard duration. Participants received no feedback during the trials.

The next trial commenced following another 1000 ms blank screen (**Figure 1**). A total of 112 randomly presented trials (each of the seven durations were presented 16 times) were completed by each participant.

### Results

Three attentional functions were calculated following the standard algorithm of Fan et al. (2002). The three attentional functions included the alerting effect, the orienting effect, and the executive control effect. To calculate the alerting effect, the mean RT of the double-cue condition was subtracted from the mean RT of the no-cue condition. A higher score indicated a larger alerting effect, which was considered to be due to the presentation of cues warning the participants of an upcoming target presentation. To calculate the orienting effect, the mean RT of the spatial-cue condition was subtracted from the mean RT of the center cue. A higher score indicated a larger orienting effect, which was considered to be due to exact spatial predictive information. To calculate the executive control effect (EC), the mean RT of the congruent flanking conditions, including all cue types, was subtracted from the mean RT of the incongruent flanking condition. A higher score indicated larger conflict interference and reduced efficiency. All data were submitted to a Pearson correlation test with analysis of the bootstrapping correlation confidence interval considering all three types of effects to assess the independence of the three attentional networks. The analysis indicated that the efficiencies of the three attentional networks were uncorrelated and therefore the scores of each network were used in subsequent analyses (**Table 1**).

**Table 1:** **Correlations between the three functions of attentional networks in Experiment 1**.

			Alerting	Orienting	EC
Orienting	Pearson correlation		-0.138	—	
	Bootstrap^c^ 95% CI	Lower	-0.408		


		Upper	0.170		
EC	Pearson correlation		-0.099	0.077	—
	Bootstrap^c^ 95% CI	Lower	-0.337	-0.183	


		Upper	0.177	0.358	
PSE (μ)	Pearson correlation		-0.379^∗^	0.220	0.027
	Bootstrap^c^ 95% CI	Lower	-0.559	-0.091	-0.250


		Upper	-0.195	0.516	0.322
SD (σ)	Pearson correlation		-0.208	0.210	0.225
	Bootstrap^c^ 95% CI	Lower	-0.341	-0.109	-0.060


		Upper	0.125	0.498	0.503

*^∗^Correlation is significant (*p* < 0.05, two-tailed).*

*^c^Bootstrap results are based on 5000 bootstrap samples.*

*NB: PSE, point of subjective equality; EC, executive control; SD, standard deviation; CI, confidence interval, respectively.*

The temporal bisection task was analyzed by computing the proportion of long duration responses for each stimulus duration. If the stimulus duration is represented as t, the proportion of long duration responses is designated as P(long| t). A plot of the proportions formed a psychometric function that is described as a sigmoid (S) curve. The start of the resulting S curve was at approximately zero, representing the shortest durations, and the end of the S curve was approximately 1, representing the longest durations. The resulting curves were fitted with a cumulative Gaussian function with a mean (μ) corresponding to the “Point of Subject Equality” and the SD (σ) corresponding to the temporal sensitivity. The Point of Subject Equality is the duration that yielded no difference between long and short responses; i.e., *P*(long| t = μ) = 0.5. A smaller σ value indicated a steeper curve and higher temporal sensitivity (Kroger-Costa et al., 2013).

We calculated the correlation of interest and established the correlation between the attentional effects and time perception by performing a Pearson correlation test with bootstrapping correlation confidence interval analysis. The results indicated a significant correlation (*p* < 0.05, two-tailed) between alerting and PSE; that is, either the lower end or upper end of the bootstrapping correlation confidence interval was either above zero or below zero, respectively. No significant correlations were found for comparisons between alerting and SD, orienting and PSE, orienting and SD, and executive control and SD (**Table 1**). Pearson correlation results were consistent with the bootstrapping correlation results. Our results suggested that alerting influenced PSE but orienting and control factors did not influence PSE.

#### Individual Differences Based on the Alerting Efficiency

To test whether individual differences in alerting efficiency influenced time perception, the participants of this study were divided into two groups; i.e., the high alerting efficiency group and the low alerting efficiency group. The median of the alerting scores (range = 0.12-98.63 ms, *Median* = 37.40 ms, SD = 18.93) was used to establish the two groups. We used an independent samples *t*-test to examine the differences in attentional functions between the two groups. Results of the *t*-test revealed a significant difference in alerting between the two groups [*t*(42) = -7.61, *p* < 0.001], whereas the differences between the two groups in orienting and executive control were not significant (|*ts*| < 1). The only significant difference between the two groups in attentional functions was in the efficiency of phasic alerting.

**FIGURE 2:**
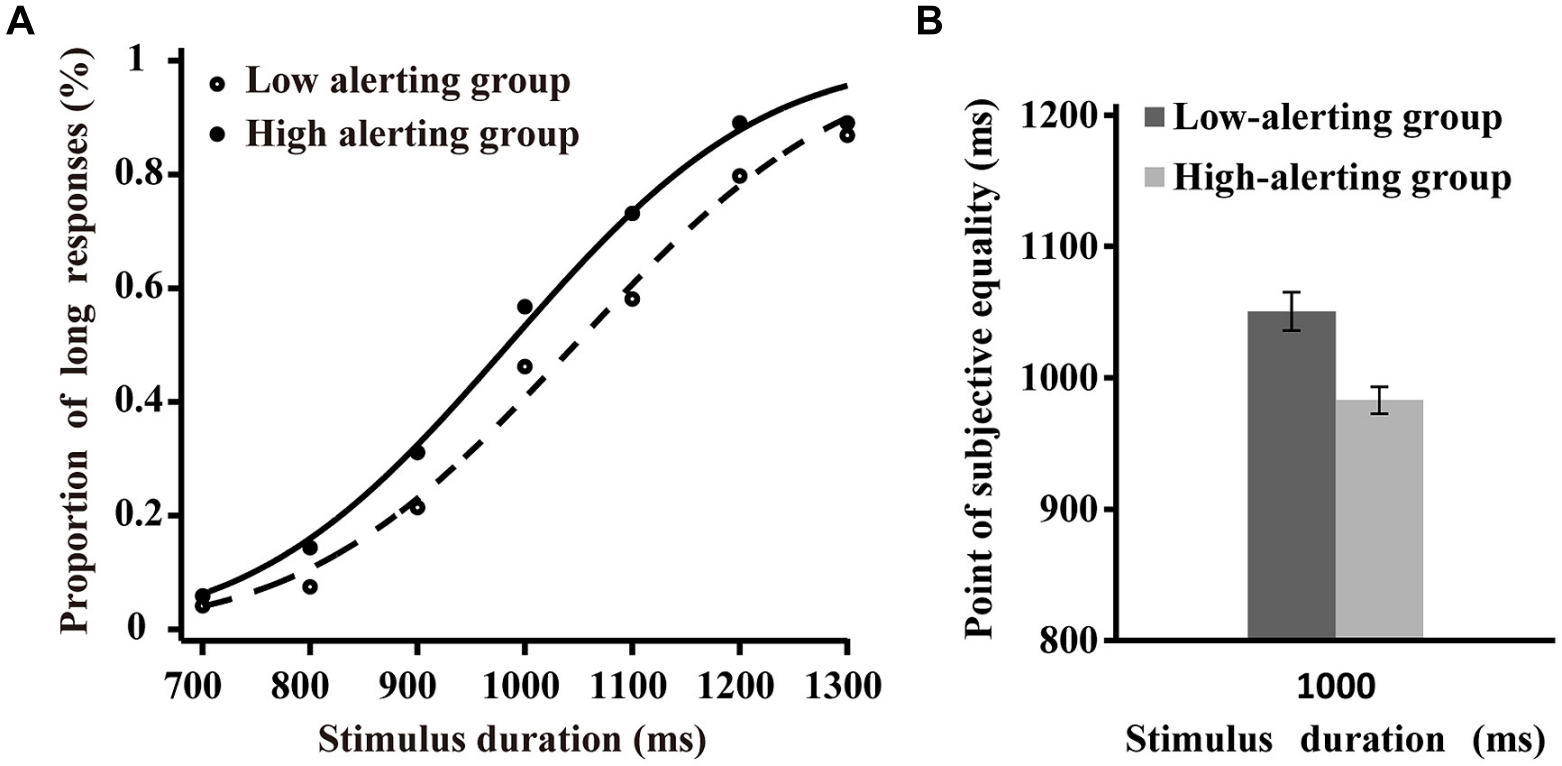
**Difference between the high alerting efficiency and low alerting efficiency groups in the temporal bisection task**. **(A)** Proportion of “Long” responses plotted as a function of stimulus duration for two groups. The lines are the best-fit cumulative Gaussian with two parameters. **(B)** Difference of the point of subjective equality between two groups.

The individual differences of time perception based on alerting efficiency were explored by comparing the estimated parameters and coefficient of determination (*R*^2^) of the psychometric functions from the low alerting efficiency and high alerting efficiency groups. The SD (σ values) ranged from 109.51 to 405.90 ms (*M* = 193.31, SD = 77.22) in the low alerting efficiency group and from 11.20 to 288.37 ms (*M* = 175.35, SD = 78.94) in the high alerting efficiency group. One-way ANOVA comparing the SDs of the two groups revealed no significant effect (*F* < 1, *p* > 0.45; **Figure 2A**). The R^2^ values ranged from 0.75 to 1.0 (*M* = 0.94, SD = 0.06) in the low alerting efficiency group and from 0.68 to 1.0 (*M* = 0.93, SD = 0.08) in the high alerting efficiency group. One-way ANOVA comparing the R^2^ values revealed no significant effect for the two groups (*F* < 1, *p* > 0.69). Individual PSEs (μ values) ranged from 939.41 to 1164.47 ms (*M* = 1050.65, SD = 67.62) in the low alerting efficiency group and from 860.72 to 1069.48 ms (*M* = 986.39, SD = 47.08) in the high alerting efficiency group. One-way ANOVA comparing the PSEs revealed significant effect for both groups [*F*(1,42) = 13.38, *p* < 0.01, η^2^ = 0.24; **Figure 2B**]. These results indicated that the alerting function of the participants could modulate subjective time but not sensitivity of time. The participants in the high alerting efficiency group tended to overestimate the physical interval whereas participants in the low alerting efficiency group tended to underestimate the physical interval.

To examine the individual differences of temporal estimation accurately based on the alerting efficiency in the temporal bisection task, we obtained a relatively accurate index of temporal estimation. The index was calculated by using the difference between PSE of estimated time and the corresponding physical time (1000 ms in current experiment), divided by the corresponding physical time. Accurate time estimates were indicated by scores close to zero. Overestimation of the target duration was indicated by positive scores and underestimation of the target duration was indicated by negative scores (Gil and Droit-Volet, 2011). The scores ranged from -0.06 to 0.16 ms (*M* = 0.05, SD = 0.07) in the low alerting efficiency group and from -0.14 to 0.07 ms (*M* = -0.01, SD = 0.05) in the high alerting efficiency group. One-way ANOVA comparing the absolute value of this score revealed significant effect [*F*(1,42) = 4.92, *p* < 0.05, η^2^ = 0.12]. These results indicated that the temporal estimations of participants in the high alerting efficiency group were more accurate than that of participants in the low alerting efficiency group.

### Experiment 2

Although previous studies have found that the results of different temporal tasks exhibited high levels of correlation (Wearden and Lejeune, 2008), cognitive processes and results of temporal judgments in the bisection and generalization task might be inconsistent (Droit-Volet and Rattat, 2007; Gil and Droit-Volet, 2011). It is necessary to verify whether the effect of alerting on time perception was stable in the generalization task. Thus, in Experiment 2, we ran the ANT and generalization tasks to investigate the influence of alerting on time perception.

#### Participants

Forty eight right-handed undergraduate and graduate students (eight males; age range = 18–25 years, mean age = 21 years) took part in the study. They all had normal or corrected-to-normal vision. All the participants signed an informed consent prior to study.

#### Procedure and Design

The ANT in Experiment 2 was similar to that in Experiment 1. The procedure for the time generalization task was similar to the bisection task (**Figure 1**). A white square on a blank screen was presented with the standard duration (1000 ms) five times. During the testing period, randomly determined durations (700, 800, 900, 1100, 1200, and 1300 ms) of the white square were presented for comparisons. The task consisted of 60 trials of standard duration and 60 trials of comparison durations (10 trials of each comparison duration), or a total of 120 trials. Each trial had similar structures as in the bisection task. The trial was initiated by presenting a fixation for 500 ms. Following the fixation point presentation, a white square target was presented in the center of the screen and then the screen went blank for a randomly determined duration of 50–1050 ms. Subsequently, the participants were instructed to estimate whether the stimulus duration was the same or not the same as the standard duration by pressing a response button. There was no feedback in these trails. The next trial commenced after another 1000 ms of blank screen. The key responses were counterbalanced between participants.

### Results

Similar to the procedure of Experiment 1, calculations were performed to determine the alerting effect, the orienting effect, and the executive control effect. Pearson correlation and bootstrapping correlation confidence interval analyses were performed to determine the independence of the three attentional networks. This analysis showed that the efficiencies of the three attentional networks were uncorrelated (**Table 2**). Therefore, the scores for each of the three attentional networks were included in subsequent analyses.

**Table 2:** **Correlations between the three functions of attentional networks in Experiment 2**.

			Alerting	Orienting	EC
Orienting	Pearson correlation		-0.137	—	
	Bootstrap^c^ 95% CI	Lower	-0.409		


		Upper	0.191		
EC	Pearson correlation		0.128	-0.093	—
	Bootstrap^c^ 95% CI	Lower	-0.160	-0.379	


		Upper	0.378	0.176	
PSE (μ)	Pearson correlation		-0.252	0.181	-0.216
	Bootstrap^c^ 95% CI	Lower	-0.487	-0.180	-0.436


		Upper	0.031	0.481	0.053
SD (σ)	Pearson correlation		-0.090	-0.109	0.103
	Bootstrap^c^ 95% CI	Lower	-0.400	-0.531	-0.150


		Upper	0.277	0.281	0.406

*^c^Bootstrap results are based on 5000 bootstrap samples.*

*NB: PSE, point of subjective equality; EC, executive control; SD, standard deviation; CI, confidence interval, respectively.*

The generalization gradients related to the proportion of “Equal” responses to stimulus duration were fitted by truncated Gaussian functions with the equation

$$y=\;\min\left\{1,\;a\,\;\;\times \;\exp\left[(-{\left(x-\mu \right)}^{2}/\left({2\sigma }^{2}\right)\right]\;\right\}$$

where *y* is the proportion of “Equal” responses, σ is the amplitude, μ is the mean (PSE), σ is the SD of the Gaussian function, and the min function, which yields an upper bound of 1, even when *a* > 1.

We also calculated the correlation of interest and established the correlation between the attentional effects and time perception by performing a Pearson correlation test with bootstrapping correlation confidence interval analysis. Results indicated no significant correlation between the data. Pearson correlation results were consistent with the bootstrapping correlation results (**Table 2**). However, the negative correlation between alerting and PSE was marginally significant (*r* = 0.252, *p* = 0.084) in Experiment 2. Our results indicated that alerting influenced PSE but orienting and control factors did not influence PSE.

#### Individual Differences Based on Alerting Efficiency

To test whether individual differences in alerting efficiency influenced time perception in Experiment 2, the participants were divided into two groups; i.e., the low efficiency alerting group and the high efficiency alerting group. The median split of the alerting scores (range = 5.02–80.11 ms, *Median* = 37.40 ms, SD = 17.30) was again used to divide the two groups. An independent samples *t*-test was used to examine the differences in attentional functions between the two groups. These results showed that the differences in alerting of the two groups were significant [*t*(46) = -7.58, *p* < 0.001], but no significant differences were observed in the orienting and executive control of the two groups (|*ts*| < 1). The results revealed that only the alerting efficiency differed in the attentional functions of the two groups.

**FIGURE 3:**
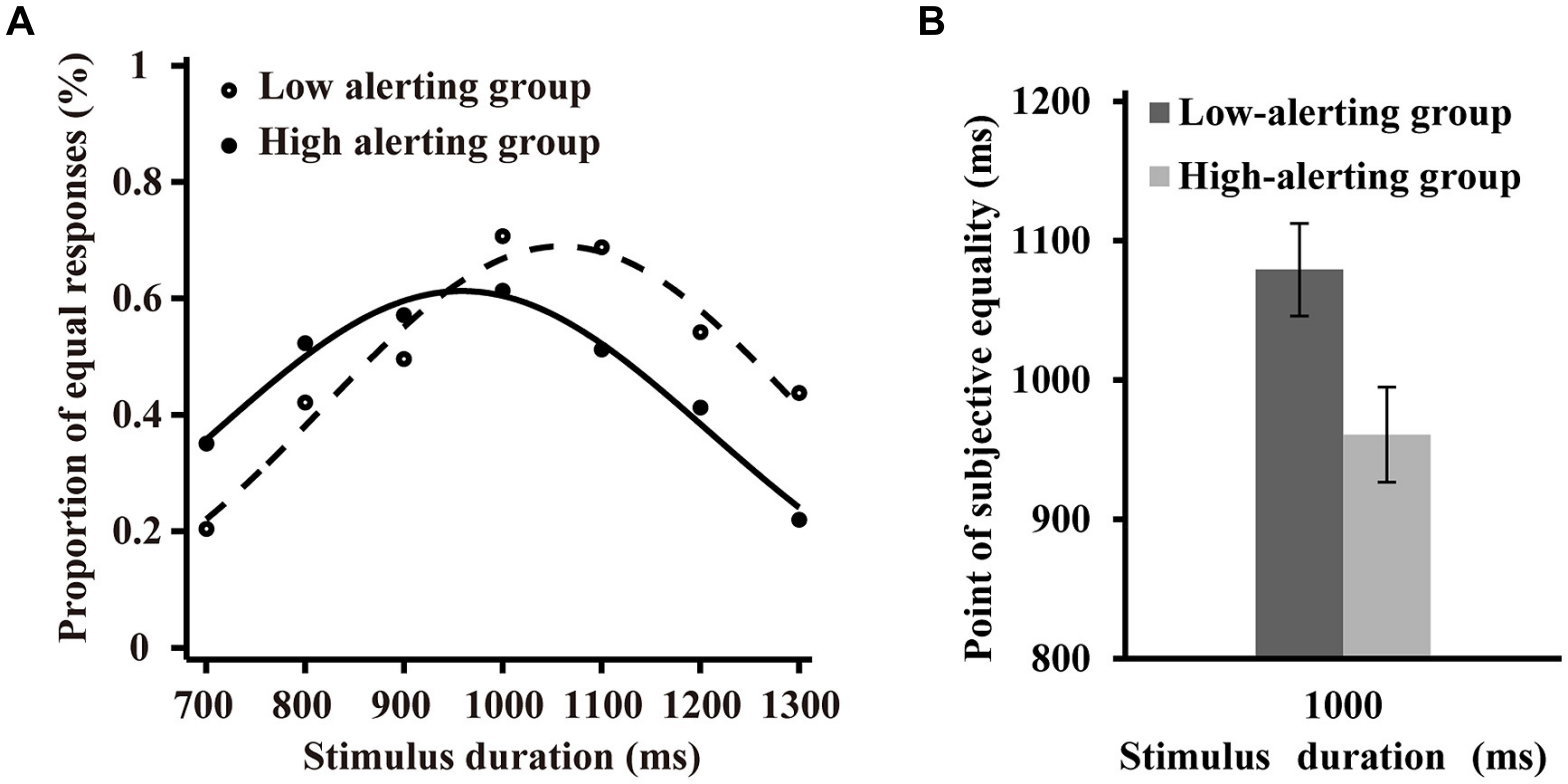
**Difference between the high alerting efficiency and low alerting efficiency groups in the time generalization task**. **(A)** Proportion of “equal” responses plotted as a function of stimulus duration for two groups. The lines are fitted by truncated Gaussian functions. **(B)** Difference of the point of subjective equality between two groups.

The estimated parameters and R^2^ of the psychometric functions of the low alerting efficiency and high alerting efficiency groups were compared to explore individual differences in time perception based on alerting efficiency. The SD (σ values) ranged from 119.03 to 443.48 ms (*M* = 216.19, SD = 69.87) in the low alerting efficiency group and from 119.18 to 335.94 ms (*M* = 206.25, SD = 46.39) in the high alerting efficiency group. One-way ANOVA comparing the SD revealed no significant effect (*F* < 1, *p* > 0.56; **Figure 3A**). The R^2^ values ranged from 0.73 to 0.98 (*M* = 0.89, SE = 0.07) in the low alerting efficiency group and from 0.72 to 0.98 (*M* = 0.89, SE = 0.07) in the high alerting efficiency group. One-way ANOVA comparing the R^2^ revealed no significant effect (*F* < 1, *p* > 0.80). Individual PSEs (μ values) ranged from 772.97 to 1475.83 ms (*M* = 1079.22, SD = 168.29) in the low alerting efficiency group and from 700.21 to 1302.99 ms (*M* = 960.82, SD = 162.55) in the high alerting efficiency group. One-way ANOVA comparing the PSEs revealed significant effects in both groups [*F*(1,46) = 6.18, *p* < 0.05, η^2^ = 0.12; **Figure 3B**]. The results were similar to those obtained in Experiment 1 and indicated that subjective time was modulated by the alerting efficiency of participants but the temporal sensitivity was not. The participants in the high alerting efficiency group tended to overestimate the physical interval whereas the participants in the low alerting efficiency tended to underestimate the physical interval.

To examine the individual differences of temporal estimation accurately based on the alerting efficiency in the generalization task, we also obtained a relatively accurate index of temporal estimation. The index was calculated using the same process as in Experiment 1. The scores ranged from -0.23 to 0.48 ms (*M* = 0.08, SD = 0.17) in the low alerting efficiency group and from -0.30 to 0.21 ms (*M* = -0.04, SD = 0.15) in the high alerting efficiency group. One-way ANOVA comparing the absolute value of this score revealed a marginally significant effect on the groups [*F* (1,46) = 3.47, *p* = 0.07, η^2^ = 0.08]. These results indicated that the temporal estimation of participants in the high alerting efficiency group might be more accurate than participants in the low alerting efficiency group.

## Discussion

This study used two kinds of time perception paradigms and found a significant negative correlation between alerting and PSE in the temporal bisection task as well as a marginally negative correlation between alerting and PSE in the generalization task. In the two experiments, participants with high alerting efficiency tended to overestimate the physical interval and estimate the duration more accurately compared with participants with low alerting efficiency. No difference in temporal sensitivity was observed between the two groups. Our study indicated that individual differences in alerting influenced time perception. We will discuss the implications of these results in the following paragraphs.

Time distortion usually results from the influence of attention and arousal in the framework of the clock model (Treisman, 1963; Meck and Church, 1983; Church, 1984; Gibbon et al., 1984; Zakay, 1989; Block and Zakay, 1996; Zakay and Block, 1996, 1997; Buhusi and Meck, 2005; Droit-Volet and Meck, 2007; Merchant et al., 2013; Shi et al., 2013). The analogical internal clock models consist of a pacemaker and an accumulator, with a switch connecting the pacemaker to the accumulator. When attention was focused on the time dimension of the stimulus, the attention-controlled switch closed, and the accumulator received the pulses from the pacemaker. The number of pulses that were accumulated determined the temporal estimation. The more pulses accumulated, the longer the subjective time was judged (Treisman, 1963; Gibbon et al., 1984).

How do the differences in alerting efficiency influence time perception through clock-like encoding? Phasic alerting is the ability which can maintain and enhance response readiness for a forthcoming stimulus through alerting events (for example, warning signals; Robertson et al., 1998; Raz et al., 2001; Raz and Buhle, 2006). Arousal is a state of general readiness (Raz and Buhle, 2006) and which can modulate the pulse speed of the pacemaker (Maricq et al., 1981; Meck, 1983). Phasic alerting can phasically increase and maintain arousal state through alerting events (Robertson et al., 1998). In all temporal tasks used in the present study, a fixation point was presented in the center of the screen at the beginning of each trial. The fixation point was presented for 500 ms and then the target square was presented. This was followed by a blank screen for a randomly determined duration of 50–1050 ms (**Figure 1**). The fixation plays a role of warning signal and prompts a temporal target to appear sequentially. Therefore, the participants with high alerting efficiency can increase and maintain higher arousal than the participants with low alerting efficiency before the temporal targets was presented. Thus, high alerting efficiency may speed up the central internal pacemaker by increasing the arousal level, which led participants to overestimate interval durations. Phasic alerting has been considered the basis for operations such as selective attention and orienting, whereas selective attention can modulate the latency of the switch that connects the pacemaker to the accumulator (Maricq et al., 1981; Meck, 1983; Droit-Volet, 2003; Husain and Rorden, 2003; Raz and Buhle, 2006; Posner, 2008). Thus, participants with high alerting efficiency may be able to open the attention-controlled switch more easily and may have shorter latency for the switch to open or close, leading to overestimation of the interval duration and a more accurate estimation of the duration compare to participants with low alerting efficiency. Therefore, based on previous studies and current findings, we can conclude that alerting efficiency may influence time perception by modulating arousal and selective attention.

Attention and arousal have different influences on the number of pulses accumulated. The attention produces an additive effect, which is a lengthening effect that remains constant regardless of the duration of the stimulus (Maricq et al., 1981; Meck, 1983). This switch latency can lead participants to overestimate interval durations and make the temporal estimation more accurately (Gil and Droit-Volet, 2011; Grommet et al., 2011). Arousal produces a multiplicative effect that can speed up the central internal pacemaker and lead to a lengthening effect. This lengthening effect is greater in longer than in shorter stimulus durations (Maricq et al., 1981; Meck, 1983). We try to dissociate the mechanisms (attention vs. arousal) underlying time performance in the current study. The results showed that participants in the high alerting efficiency group tended to overestimate the physical interval and estimate duration more accurately compared to participants in the low alerting efficiency group, whereas no significant differences were observed in the SD (steeper curves and higher temporal sensitivities are associated with smaller σ values) between the two groups. These findings appear to result from the effect of the attention-controlled switch. However, we admit that we encountered conflicts on whether to use one but not several sets of time durations to distinguish the effects of attention or arousal. For example, some issues may be encountered when submitting data on the proportion of “Long” responses to calculate the difference in the slope of the fitted curve, because the proportion of “Long” responses in some durations may have a ceiling effect. Thus, to distinguish between effects of attention or arousal, future studies should use not only one but several sets of time durations to dissociate the mechanisms. However, according to previous alerting-related studies as well as related studies on time distortion, we infer that alerting efficiency can influence temporal estimation by modulating the arousal and selective attention.

From another perspective, previous studies demonstrated that subjective time dilation was a global visual experience (New and Scholl, 2009). Phasic alerting can enhance global processing of visual items and increase attention to perceptual stimuli in the circumstances, allowing participants with high alerting efficiency to better use the alert signal (Weinbach and Henik, 2011). These participants were also better at global processing, thereby leading to time dilation. Thus, we speculate that the individual alerting efficiency could also influence time perception by enhancing the global processing.

Most importantly, the neuroimaging studies have revealed that neural correlates of phasic alerting were important in the pre-supplementary motor area (pre-SMA). Phasic alerting can be modulated by the potentiation of the pre-SMA through the midbrain–thalamus–anterior cingulate cortex (ACC) alerting network (Yanaka et al., 2010; Yoshida et al., 2013). A wealth of evidence suggests that pre-SMA plays a key role in time processing, and can be a neural substrate of the accumulator to support accumulation and maintenance of temporal information (Coull et al., 2004; Casini and Vidal, 2011; Schwartze et al., 2012; Merchant et al., 2013; Tipples et al., 2013; Herrmann et al., 2014). Therefore, the significant correlation between alerting and temporal estimation may be due to sharing common processes with related mechanisms of pre-SMA.

## Conclusion

This study used the ANT task and two kinds of time perception paradigms to explore the influence of alerting on time perception. The results showed that participants with high alerting efficiency tended to overestimate the physical interval and estimated the duration more accurately compared with participants with low alerting efficiency. No differences in the sensitivity of time were observed between the two groups. According to previous studies and the current findings, we infer that individual differences in alerting might influence time perception through the attention-controlled switch and the speed of the pacemaker. Actually, both high alerting efficiency and temporal overestimation enable individuals to adapt to events efficiently in their environment.

## Conflict of Interest Statement

The authors declare that the research was conducted in the absence of any commercial or financial relationships that could be construed as a potential conflict of interest.
